# Supplementary material for: Potential role of photobiomodulation as a prevention and treatment strategy for radiation induced fibrosis: a review of effectiveness and mechanisms
Source: PeerJ. 2025 Jun 2;13:e19494. doi: 10.7717/peerj.19494 (PMC12139614; doi:10.7717/peerj.19494)
Supplement: Supplemental Information 2 [file peerj-13-19494-s002.docx]

Online resource1: Detailed search terms and the search strategy used across databases

**Search Terms**

| RIF Terms | PBMT Terms |
| --- | --- |
| Radiation induced fibrosis  Radiation-induced fibrosis  Radiation fibrosis syndrome  Chronic radiation injury  Radiotherapy fibrosis | Photobiomodulation  Low level laser therapy  Low-level laser therapy  Low level light therapy  Low-level light therapy  Low power light therapy  Low-power light therapy  Light-emitting diode  Light emitting diode  Red light  Infrared light  Phototherapy  Biostimulation  PBMT  LLLT  LED |

**Search strategy across databases**

**PubMed** 680

#1

(((((Radiation fibrosis syndrome [MeSH]) OR (Radiation induced fibrosis)) OR (Radiation-induced fibrosis)) OR (Radiation fibrosis syndrome)) OR (Chronic radiation injury)) OR (Radiotherapy fibrosis)

#2

((((((((((((((((Photobiomodulation) OR (Low level laser therapy)) OR (Low-level laser therapy)) OR (Low level light therapy)) OR (Low-level light therapy)) OR (Low power light therapy)) OR (Low-power light therapy)) OR (Light-emitting diode)) OR (Light emitting diode)) OR (Red light)) OR (Infrared light)) OR (Phototherapy)) OR (Biostimulation)) OR (PBMT)) OR (LLLT)) OR (LED)) OR (Low-level light therapy [MeSH])

#1 AND #2 680

**LILACS (497)**

((Radiation induced fibrosis ) OR (Radiation-induced fibrosis) OR (Radiation fibrosis syndrome) OR ( Chronic radiation injury ) OR (Radiotherapy fibrosis)) AND ((Photobiomodulation) OR ( Low level laser therapy) OR ( Low-level laser therapy ) OR (Low level light therapy ) OR (Low-level light therapy ) OR (Low power light therapy ) OR (Low-power light therapy ) OR (Light-emitting diode ) OR (Light emitting diode ) OR (Red light ) OR (Infrared light ) OR (phototherapy) OR (biostimulation) OR (PBMT ) OR (LLLT ) OR (LED))

**Scopus (411)**

( ( TITLE-ABS-KEY ( radiation AND induced AND fibrosis ) OR TITLE-ABS-KEY ( radiation-induced AND fibrosis ) OR TITLE-ABS-KEY ( radiation AND fibrosis AND syndrome ) OR TITLE-ABS-KEY ( chronic AND radiation AND fibrosis ) OR TITLE-ABS-KEY ( radiotherapy AND fibrosis ) OR TITLE-ABS-KEY ( "radiation induced fibrosis" ) OR TITLE-ABS-KEY ( "radiation fibrosis syndrome" ) OR TITLE-ABS-KEY ( "chronic radiation fibrosis" ) OR TITLE-ABS-KEY ( "radiation-induced fibrosis" ) ) ) AND ( ( TITLE-ABS-KEY ( photobiomodulation ) OR TITLE-ABS-KEY ( pbmt ) OR TITLE-ABS-KEY ( low AND level AND laser AND therapy ) OR TITLE-ABS-KEY ( low AND level AND light AND therapy ) OR TITLE-ABS-KEY ( lllt ) OR TITLE-ABS-KEY ( led ) OR TITLE-ABS-KEY ( light-emitting AND diode ) OR TITLE-ABS-KEY ( low-level AND light AND therapy ) OR TITLE-ABS-KEY ( low AND power AND laser AND therapy ) OR TITLE-ABS-KEY ( phototherapy ) OR TITLE-ABS-KEY ( biostimulation ) OR TITLE-ABS-KEY ( "low level laser therapy" ) OR TITLE-ABS-KEY ( "low level light therapy" ) OR TITLE-ABS-KEY ( "light-emitting diode" ) ) )

**EBSCO (217)**

S1

Radiation induced fibrosis OR Radiation-induced fibrosis OR Radiation fibrosis syndrome OR Chronic radiation injury OR Radiotherapy fibrosis

S2

Photobiomodulation OR Low level laser therapy OR Low-level laser therapy OR Low level light therapy OR low-level light therapy OR Low power light therapy OR Low-power light therapy OR Light-emitting diode OR Light emitting diode OR Red light OR Infrared light OR Phototherapy

S3

Biostimulation OR PBMT OR LLLT OR LED

S4

S2 OR S3

S5

S1 AND S4

**ProQuest 926**

((((abstract(Radiation induced fibrosis) OR abstract(Radiation-induced fibrosis) OR abstract(Radiation fibrosis syndrome) OR abstract(Chronic radiation injury) OR (Radiotherapy fibrosis)) AND peer(yes)) AND PEER(yes)) AND ((((abstract(Infrared light) OR abstract(Phototherapy) OR abstract(Biostimulation) OR abstract(PBMT) OR abstract(LLLT) OR abstract(LED)) AND peer(yes)) AND PEER(yes)) OR (((abstract(Photobiomodulation) OR abstract(Low level laser therapy) OR abstract(Low-level laser therapy) OR abstract(Low level light therapy) OR abstract(Low-level light therapy) OR abstract(Low power light therapy) OR abstract(Low-power light therapy) OR abstract(Light-emitting diode) OR abstract(Light emitting diode) OR abstract(Red light)) AND peer(yes)) AND PEER(yes)))) AND PEER(yes)
